# Penile vascular macules: an under-recognised condition responding to pulsed dye laser

**DOI:** 10.1007/s10103-025-04695-2

**Published:** 2025-10-22

**Authors:** Vishal Madan

**Affiliations:** 1 Dermatology, Everything Skin Clinic™, High Street, Cheadle, SK8 1AL Manchester, UK; 2 University of Bolton, Bolton, UK

## Abstract

Penile vascular macules represent an under-recognised clinical entity that can present both idiopathically and secondary to trauma. These lesions, while benign, often cause significant psychological distress to patients. Three cases of Penile vascular macules with distinct aetiologies are presented. Two cases demonstrated idiopathic onset during adolescence, presenting as solitary, asymptomatic red macules on the glans penis. The third case was acquired, developing secondary to prolonged penis ring use. The idiopathic cases showed complete resolution with pulsed dye laser treatment with sustained results at 12-month follow-up, while the acquired case declined intervention. Recognition of Penile vascular macules as a distinct clinical entity is crucial for appropriate patient counselling and management. While benign, these lesions can cause a significant psychological impact, and treatment options, including pulsed dye laser and alternative modalities, should be discussed with affected patients.

## Introduction

Asymptomatic red macules on the glans penis are rare and may cause anxiety in patients due to cosmetic concerns. These macules can be idiopathic, appear spontaneously, or are acquired following trauma. The sparse literature on this topic necessitates a better understanding of these presentations to guide clinical practice. This report describes three cases of penile vascular macules (PVMs)—two idiopathic and one acquired—suggesting that these may represent a distinct clinical entity with specific pathophysiological mechanisms.

## Case series

### Case 1

**Fig. 1:**
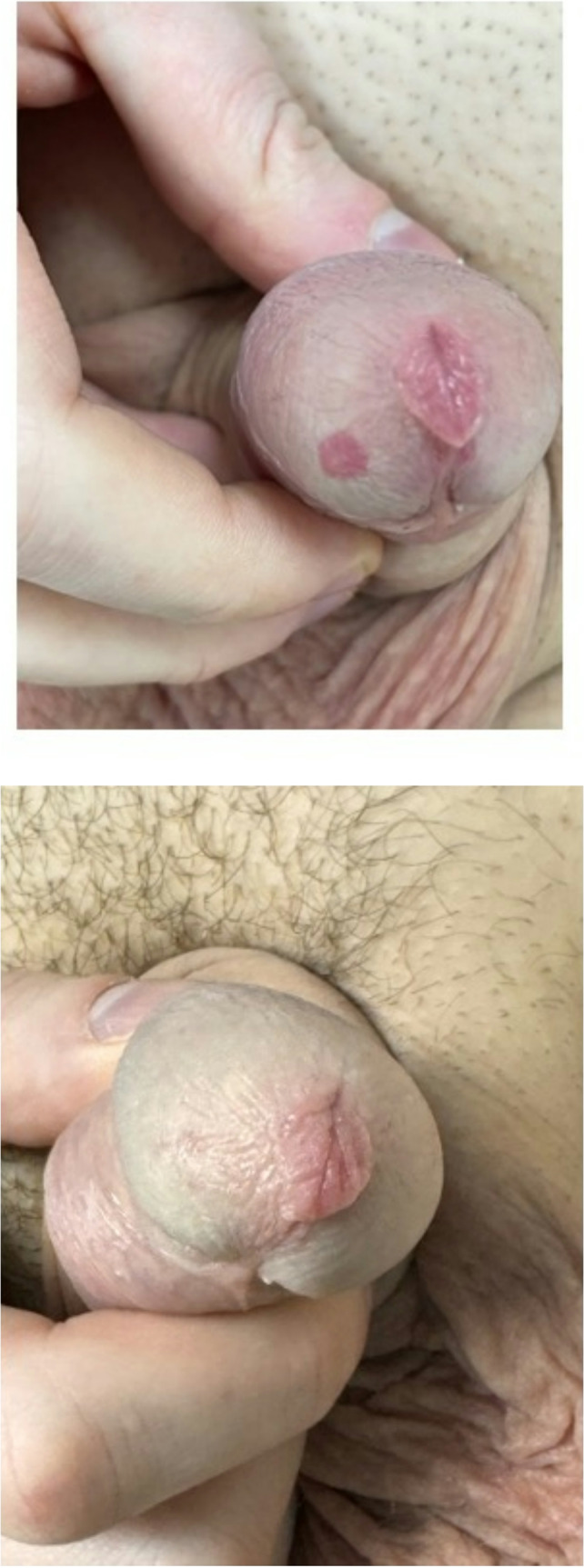
Penile vascular macule in Case 1 (**a**) Before and (**b**) After Pulsed dye laser treatment

A 24-year-old male presented with a 10-year history of a solitary, painless red macule on the glans penis (Fig. 1a). The lesion had remained stable in size and appearance over the years. The patient denied any history of trauma, sexual activity before the appearance of the macule, or other relevant medical history. On examination, a 5 mm vascular macule was noted on the glans penis, which blanched upon diascopy. The lesion was of cosmetic concern to the patient, though it was asymptomatic and benign. He underwent three sessions of pulsed dye laser (PDL, Candela V Beam Prima, 595 nm, 7 mm spot, 8–9 J/cm², DCD: 30/20), resulting in complete resolution of the lesion (Fig. 1b). At 12-month follow-up, there was no recurrence of the lesion, and the patient reported high satisfaction with the cosmetic outcome.

### Case 2

**Fig. 2:**
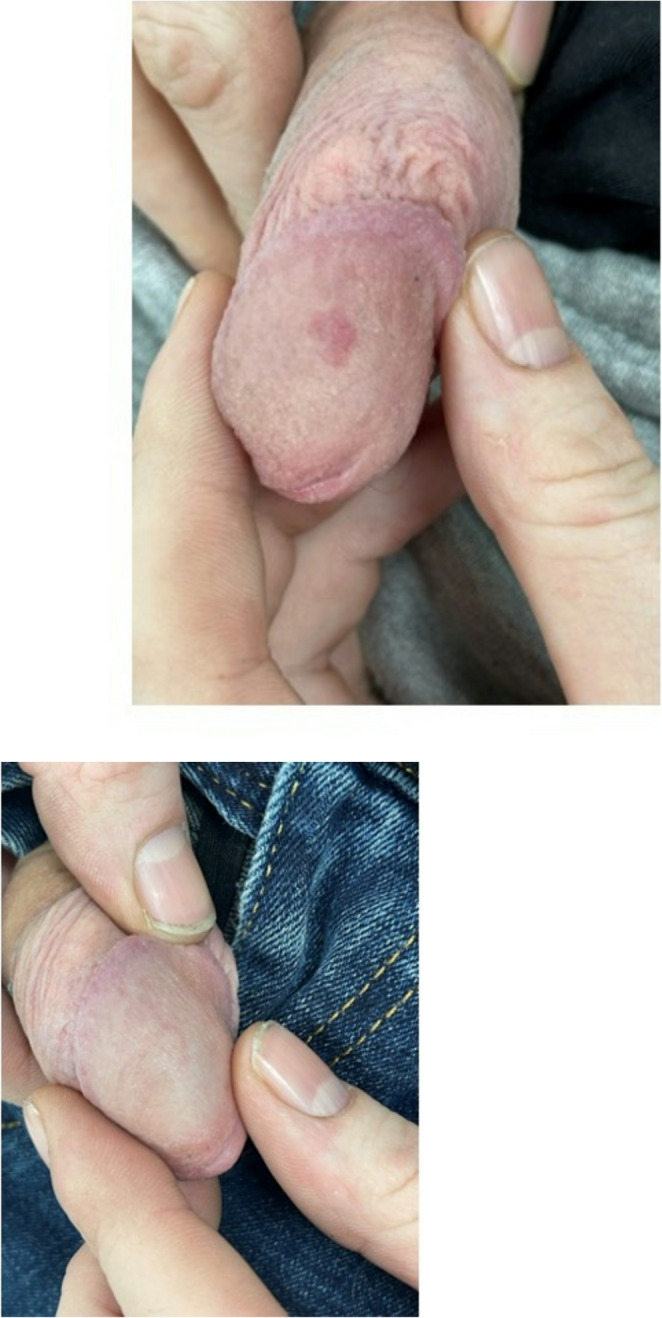
Penile vascular macule in Case 2 (**a**) Before and (**b**) After Pulsed dye laser treatment

 A 30-year-old man noticed a red macule on his glans penis (Fig. 2a) during his teenage years. The lesion appeared spontaneously, without preceding trauma or sexual activity. Over the years, it persisted, causing significant sexual anxiety. On examination, the lesion was a blanching vascular macule, measuring 6 mm in diameter. The patient underwent 2 PDL treatments, which yielded excellent results (Fig. 2b). Follow-up at 6 months and 18 months post-treatment confirmed persistent clearance with no signs of recurrence.

### Case 3

**Fig. 3:**
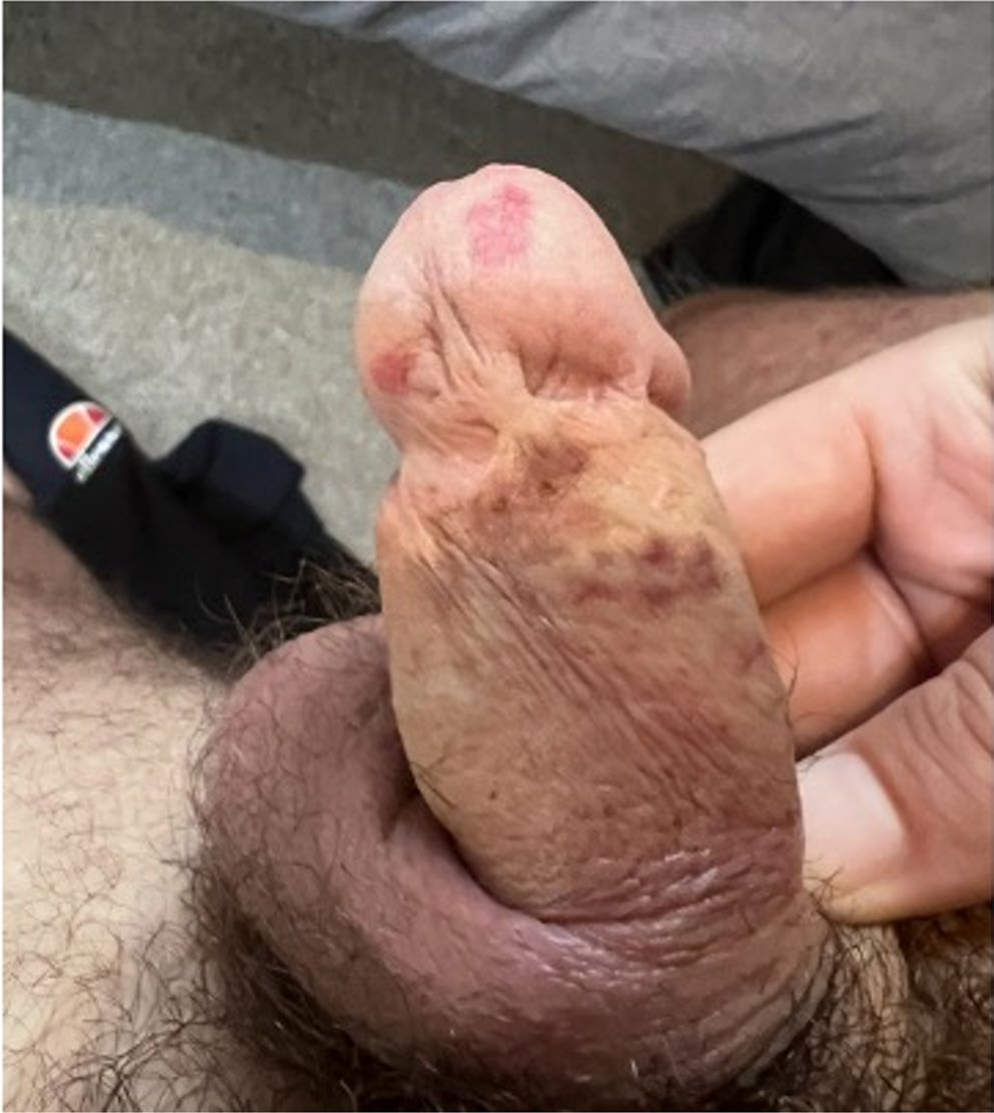
Vascular Macules on glans penis of Case 3. Hyperpigmentation on the skin for penile shaft from chronic friction from penis ring is also seen

 A 45-year-old circumcised man presented with red marks on his glans penis (Fig. 3) that emerged after prolonged and excessive use of a penis ring—several hours daily for four years. Although asymptomatic, these marks severely adversely affected his personal life. Additionally, he developed dark spots on the shaft of his penis from friction caused by the penis ring. Despite these concerns, the patient opted against laser treatment. The patient was provided with comprehensive education regarding safe penis ring use and advised to discontinue usage for at least 3 months to assess for spontaneous resolution.

## Discussion

PMVs are uncommon or likely under-reported. The cases described in this report suggest that these lesions can occur idiopathically or following trauma. The benign nature of the macules, evidenced by the lack of progression over the years, suggests that they may be managed conservatively, especially when asymptomatic. Reassurance should be provided regarding the benign nature of the condition; however, addressing the patient’s cosmetic concerns is also crucial.

PVMs likely represent localised vascular ectasias, characterised by dilatation of superficial dermal blood vessels. The pathophysiology differs between idiopathic and acquired cases:

### Idiopathic PVMs

In spontaneous cases, developmental vascular anomalies, akin to those in other cutaneous vascular ectasias, may be incriminated. These may stem from localised defects in vascular tone regulation, influenced by hormonal changes during puberty, as seen in adolescent idiopathic cases. Histologically, these lesions likely consist of dilated capillaries in the papillary dermis without evidence of neovascularisation.

### Acquired PVMS

In traumatic cases, mechanical stress may cause microvascular damage. Specifically, prolonged constriction from penis rings leads to venous engorgement and increased intracorporeal pressure, resulting in microvascular rupture or persistent dilatation. This mechanism resembles purpuric lesions, but the blanching nature of PVMs suggests persistent vascular dilatation rather than erythrocyte extravasation. Chronic friction may also stimulate post-inflammatory angiogenesis, contributing to vascular prominence.

These pathophysiological differences may result in differential treatment responses between idiopathic and acquired PVMs. Idiopathic lesions may respond well to targeted vascular destruction via PDL, while acquired lesions might require removal of the inciting factor for resolution.

The differential diagnoses include angiokeratomas, Zoon’s balanitis, and other vascular lesions. PVM can be differentiated from other penile vascular lesions through a thorough clinical examination (Table 1). Genital purpura can be the presenting feature of lichen sclerosus [2]. However, unlike PMV these present as purple, non-blanching and irregularly bordered macules. The blanching observed on diascopy supports a vascular aetiology in PMV.

**Table 1:** Differential diagnoses of penile vascular macules and differentiating features [1]

Feature	PVMs	Angiokeratomas	Zoon’s Balanitis	Genital Purpura (Lichen Sclerosus)
Colour	Bright red	Dark red-purple	Shiny red-orange	Purple
Border	Well-defined	Irregular	Poorly defined	Irregular
Surface	Flat	Elevated	Glazed	Flat
Blanching	Present	Absent	Partial	Absent
Distribution	Solitary/Few	Multiple	Patches	

Penis rings, also known as cock rings, are devices worn around the base of the penis, often used to enhance sexual performance by restricting blood flow, which may prolong erection [3]. However, the use of such devices can lead to various complications, including vascular injury, which might present as localized erythema, bruising, or in some cases, necrosis from strangulation [4, 5]. The association of the lesion in Case 3 with the use of a penis ring underscores the potential for trauma-induced vascular changes in the penis, particularly when worn for extended periods or applied too tightly. The mechanism of injury typically involves constriction of venous outflow, leading to increased intracorporeal pressure [5]. This can result in vascular trauma manifesting as persistent erythema, bruising, or the formation of vascular macules, as noted in Case 3. The use of penis rings should be considered a potential risk factor for PVMs, particularly in cases where there is no other apparent cause for the lesion. Although these complications are relatively rare, they underscore the importance of patient education on the safe use of such devices. Vascular lesions on the penis following trauma have been described.

A case of recurring petechia on the glans penis from capillary rupture, secondary to fellatio in an adult man who was on treatment with acetylsalicylic acid has been reported [6].

These cases highlight the variability in presentations associated with penile vascular macules. Case 2, like the first, involved idiopathic onset during the teenage years, while Case 3 involved trauma associated with the use of a penis ring, consistent with findings in the literature regarding penile ring-induced vascular changes.

While idiopathic PVMs readily responded to PDL, the natural history of acquired PVMs is uncertain. Like idiopathic PVMs, these should respond to vascular-specific lasers, however, spontaneous resolution if trauma is avoided is possible.

While PDL was the treatment modality used in idiopathic PVMs in the current series, alternative therapeutic options may include:

1. *Intense Pulsed Light (IPL)*: IPLs emit polychromatic light at various wavelengths (515–1200 nm) and may be effective for superficial vascular lesions with potentially less purpura than PDL.
2. *Long-pulsed Nd: YAG laser (1064 nm)*: This may be suitable for deeper vascular lesions and in patients with darker skin types. However, caution should be exercised in genital applications due to deeper penetration and an increased risk of scarring.
3. *Topical treatments*: Topical vitamin K or tranexamic acid preparations for patients declining laser intervention may reduce vascular prominence, though evidence for efficacy in PVMs is lacking.
4. *Cryotherapy*: In resource-limited settings, gentle cryotherapy might be considered, though risk of hypopigmentation and scarring in genital tissues must be carefully weighed.
5. *Electrodessication*: A cautious application for very small lesions could be considered, though the scarring risk is higher than with vascular-specific lasers.

In cases of acquired PVMs, like Case 3, the main management approach focuses on eliminating the triggering factor. This entails stopping the use of a penis ring and educating the patient on the appropriate use of sexual enhancement devices. Key points include limiting how long the device is worn, ensuring it fits correctly, removing it immediately if the patient feels pain, numbness, skin colour changes, or swelling, and using adequate lubrication.

Future studies might help establish the prevalence of these lesions and explore any potential associations with other dermatological conditions or practices, such as the use of penis rings. This is particularly important as the popularity of such devices may increase, potentially leading to a rise in trauma-related penile lesions.

## Conclusion

PVMs, whether idiopathic or acquired, represent a benign but potentially distressing condition for patients and are likely more common than reported. Clinicians should be aware of this entity to provide accurate diagnosis and management, alleviating patient anxiety. Special attention should be paid to the use of devices such as penis rings, which may contribute to the development of acquired PVMs.

While PDL offers a safe and reliable treatment option for idiopathic PVMs with demonstrable long-term efficacy, alternative treatment modalities may be considered based on availability and patient preference. For acquired PVMs, removal of the inciting factor, coupled with comprehensive patient education on safe practices, forms the cornerstone of management. The distinct pathophysiological mechanisms underlying idiopathic versus acquired PVMs likely explain the differences in clinical presentation and treatment response, highlighting the importance of distinguishing between these subtypes in clinical practice.

## Data Availability

No datasets were generated or analysed during the current study.

## References

[1] Bunker CB (2010) Skin conditions of the male genitalia. Medicine 38:294–299

[2] Lewis M, Mercurio MG (2016) Genital purpura as the presenting sign of lichen sclerosus. J Am Acad Dermatol 74:e97–e98 27085248 10.1016/j.jaad.2015.12.005

[3] WebMD E Team (n.d) Cock Rings: What You Need to Know. [online] WebMD. Available at: https://www.webmd.com/sex/cock-rings

[4] Forrester MB (2022) Penis ring injuries treated at emergency departments. J Sex Marital Ther 48:103–111 33734034 10.1080/0092623X.2021.1900003

[5] Harris E, Llompart D, Izquierdo G, Aziz MA (2020) Patient with penile and scrotal strangulation due to prolonged use of a metal ring device. Cureus 12:5 10.7759/cureus.11928 PMC7785473 33425509

[6] Marasca C, Cappello M, Patruno C, Marasca D, Squillace L, Megna M (2019) Petechia of the penis: sexual habits or adverse drug reaction? Curr urol. 12:167–168 31316327 10.1159/000489438 PMC6613315

